# The Presence of Predatory and Open Access Journal Publications Among Canadian Plastic Surgery Residency Applicants

**DOI:** 10.1177/22925503241300336

**Published:** 2024-12-09

**Authors:** Adrianna Keeler, Hassan ElHawary, Young Ji Tuen, Rebecca Courtemanche, Mirko S. Gilardino, Jugpal S. Arneja

**Affiliations:** 1 Faculty of Medicine, University of British Columbia, Vancouver, BC, Canada; 2 Division of Plastic Reconstructive and Aesthetic Surgery, 54473 McGill University Health Centre, Montreal, QC, Canada; 3 Division of Plastic Surgery, University of British Columbia, Vancouver, BC, Canada

**Keywords:** predatory journals, open-access journal, research integrity, plastic surgery, medical student, revues prédatrices, journaux en accès libre, intégrité de la recherche, chirurgie plastique, étudiants en médecine

## Abstract

**Introduction:** As scientific publishing has transitioned online, open access and predatory publishers have surged. This study describes the frequency of publications in potentially predatory and open access journals among applicants to a Canadian plastic surgery residency program, and explores applicant characteristics associated with open access and predatory publishing. **Methods:** A retrospective review of plastic surgery resident applicants’ curriculum vitae (CVs) from 2015 to 2018 was performed. Published articles listed in CVs were reviewed by 2 authors to identify publication availability, publication year, and publisher. Open access publications were identified using the Directory of Open Access Journals. Predatory publications were identified using Beall's list of potentially predatory publishers. Published applicants’ characteristics were summarized. Applicant characteristics associated with open access and predatory publishing were explored using logistic regression. **Results:** Of the 186 applicants, 117 published 388 articles and were included in the final analysis. 156 (40.2%) articles were published in open access journals by 76 (40.8%) applicants. 14 (3.6%) articles were published in predatory journals by 14 (7.5%) applicants. Applicant characteristics associated with open access publishing included total number of publications (OR: 1.56, 1.18-1.93, *P* < .001) and presence of at least one post-baccalaureate degree (OR: 0.36, 0.13-0.95, *P* = .038). Only an applicant's total number of publications (OR: 1.25, 1.06-1.48, *P* = .010) was significantly associated with publishing in a predatory journal. **Conclusion:** These findings stress the importance of raising awareness within the plastic surgery community, including medical students, about the deceptive nature of predatory journals.

## Résumé

**Introduction :** Avec la transition vers la publication scientifique en ligne, les options de revues prédatrices et en accès ouvert ont explosé. Cette étude décrit la fréquence des publications dans des journaux en accès ouvert et dans des revues potentiellement prédatrices parmi les candidats à des programmes canadiens de résidence en chirurgie plastique et elle a exploré les caractéristiques des candidats associés à ces publications. **Méthodes :** Nous avons mené une revue rétrospectives des curriculum vitae (CV) des candidats à un programme de résidence en chirurgie plastique de 2015 à 2018. Les articles publiés énumérés dans les CV ont été revus par deux auteurs pour identifier la disponibilité et l’année de la publication, ainsi que l’éditeur. Les publications en accès libre ont été identifiées au moyen du Directory of Open Access Journals. Les publications prédatrices ont été identifiées en utilisant la liste de Beall des éditeurs potentiellement prédateurs. Les caractéristiques des candidats publiés ont été résumées. Les caractéristiques des candidats associées à des publications dans des revues prédatrices et en accès libre ont été explorées au moyen d’une régression logistique. **Résultats :** Parmi les 186 candidats, 117 ont publié 388 articles et ont été inclus dans l’analyse finale. Parmi les articles, 156 (40,2 %) ont été publiés dans des revues en accès ouvert par 76 candidats (40,8 %) et 14 articles (3,6 %) ont été publiés dans des revues prédatrices par 14 candidats (7,5 %). Les caractéristiques des candidats associés à des publications en accès ouvert ont inclus le nombre total de publications (RC : 1,56; 1,18 à 1,93; *P* < ,001) et la présence d’au moins un diplôme post baccalauréat (RC : 0,36; 0,13 à 0,95; *P* = ,038). Seul un nombre total de publications du candidat (RC : 1,25; 1,06 à 1,48; *P* = ,010) a été associé de manière significative à une publication dans une revue prédatrice. **Conclusion :** Ces constatations soulignent l’importance de la sensibilisation de la communauté de la chirurgie plastique, y compris des étudiants en médecine, sur la nature trompeuse des revues prédatrices.

## Introduction

Scientific publishing has undergone a significant transition over the last few decades from print-based, subscription access journals mainly targeted towards a select community of physicians and scientists to the broadly accessible open access model that is primarily online.^1^ One of the main goals of open access publishing is to improve the public's access and understanding of scientific evidence and publicly funded research. Between 2013 and 2023 the percent of global research articles, reviews, and conference papers published in open-access journals increased from 11% to 38%.^2^ Despite the many advantages of open access research, this format has paved the way for predatory journals to arise, which poses a fundamental threat to the peer review process and the integrity of research.

Predatory journals are defined as those that exploit the open access model of scientific publishing to profit from scholarly publishing in a dishonest and unprofessional manner.^3–5^ These journals often engage in unethical practices; these practices typically involve promising rapid peer review while having a false or fabricated editorial process with the fundamental goal of making a profit from researchers. They pose a danger to the key aspects of scholarly communication and take advantage of the open access publishing model, which has many benefits to both the author and the reader.^3^

Currently, there is a paucity of evidence surrounding the prevalence of open access publishing practices among plastic surgery applicants, and how this compares to the overall prevalence in scientific publishing. Additionally, previous research has shown that the majority of authors that publish in predatory journals are young, inexperienced, and often from middle- or low-income countries.^6,7^ Investigation of this phenomenon among general surgery medical trainees identified that approximately 1% had published in a predatory journal.^8^ While previous papers have investigated the prevalence and characteristics of predatory journals in plastic surgery,^9,10^ there is a paucity of evidence with regards to the presence of predatory journal publishing habits specifically among plastic surgery residency applicants. Therefore, this study aimed to describe the prevalence of predatory journal and open access journal publications among plastic surgery residency applicants and identify any applicant characteristics associated with publishing in open access publications and predatory journals.

## Methods

This study was designed as a retrospective review of residency applicants’ curriculum vitae (CVs) submitted to McGill University's Plastic Surgery residency program for the 2015-2018 academic years. Data was obtained from a dataset previously analyzed by Elhawary et al.^11^ All the identifying information was concealed from all the reviewers that were involved in the data extraction and analysis to maintain the applicants’ confidentiality. Ethics approval was obtained from the McGill University Research Ethics Board (A03-B15-23B) and the University of British Columbia Children and Women’s Clinical Research Ethics Board (H22-03753).

Applicants were reviewed for repeat applications over the 4-year time period. For those that applied more than once, only the CV submitted in the final year of application was included in the study to allow for the most complete report of publications. Applicant characteristics were collected: year of application, gender, region of medical education, post-baccalaureate degree(s), average impact factor of publications, and total number of publications.

Articles listed on applicant CVs were reviewed independently by two authors (A.P. and Y.J.T.). Duplicate publications on a single applicants’ CV were excluded. Each article citation (status “published,” “accepted,” or “in-press”) was searched on PubMed, Web of Science, and Google Scholar. Articles not found were then searched on the journal publication's website, and those not found were excluded. For articles included, the corresponding journal's publishing company, impact factor, and open access or predatory status was obtained according to Table 1. Articles identified as being published in a predatory journal were reviewed to identify length of time from submission to acceptance, alignment of article content with journal topic, and if the article was related to plastic surgery.

**Table 1.** Journal Factors.

Factor	Source of information
Publishing company	Scopus Source List or Journal Website^12^
Impact factor	Clarivate Journal Citation Report^13^ (If journal was not found, an impact factor of 0 was assigned)
Open access	Directory of Open Access Journals^14^ or Journal Website
Predatory	Beall's List of Potential Predatory Journal and Publishers, 2021^15^

### Analysis

Applicant characteristics were tabulated by whether they had one or more publications in a predatory journal. Continuous variables with normal distribution were summarized with means and standard deviations or medians and interquartile ranges (IQR), while categorical variables were summarized with counts and percentages. The characteristics of applicants with none versus one or more publications in a predatory journal were compared using univariate analyses; Chi-squared tests for categorical variables, or Mann–Whitney–Wilcoxon tests for continuous variables when investigation with a preliminary Shapiro–Wilk test identified these variables as not normally distributed. The association between residency applicant characteristics with one more open access publications, or one or more predatory publications was further explored using logistic regression using a purposeful selection model building approach to favor selection of risk factors, rather than to generate a predictive model.^13^ In short, applicant characteristics including year of application (continuous), medical school region (Canadian, US, Other), male gender, post-baccalaureate degree (binary), average impact factor of applicants publications (continuous), and number of publications by applicant (continuous) were assessed in a bivariate regression and retained in the preliminary model at an alpha value ≤0.25.^13^ In the iterative process of variable selection, covariates were removed from the preliminary model if they had a *P*-value ≥.10 and were not found to be a confounder of the significant covariates (>20% change in the coefficient).^13^ Variables not selected in the first step were re-introduced into the preliminary model one by one, and retained if they were significant at the 0.10 level.^13^ Statistical significance is reported at an alpha value of 0.05. All analyses were performed using R version 4.3.0.^16^

## Results

### Applicant Characteristics

Across the span of the 2015-2018 residency application cycles, 196 applications were submitted to McGill University's Plastic Surgery Residency Program. Of these applicants, 8 (4.3%) applied in multiple cycles, leaving 186 unique applicants. Overall, 117 (63%) applicants were confirmed to have authored 388 research articles in 255 scientific journals. Among published applicants, the median number of published journal articles was 2 [IQR: 1,4]. The median impact factor of all publications was 2.1 [0.65, 3.46]. In total, 156 (40.2%) articles were published in open access journals by 76 (40.8%) applicants.

### Predatory Publications

Among confirmed publications, 14/388 (3.6%) articles were published in 11 predatory journals by 14/196 (7.1%) applicants. Predatory publications represented 14/156 (8.9%) of all open access publications. All articles published in predatory journals were available online, other than one, which only had an available abstract. Publication dates ranged from 2012 to 2017. All 11 predatory journals listed a continuous publishing model. The mean time from submission to acceptance and from acceptance to publication listed on each article was 49 days (Min: 8 days; Max: 195 days) and 70 days (Min: 0 days; Max: 214 days), respectively. Research topics varied widely across publications and matched the journal topics. Of note, 3/14 (21.4%) predatory publications were in a journal focused on plastic surgery. Further review of the predatory journals identified 2 that advertised a “time to acceptance” of less than 7 days on their home page, and one noted a “first-pay, first-publish” policy. All articles published in predatory journals that advertised an expedited review processes were published in the year preceding the application cycle of the applicant. Characteristics of predatory publications are listed in Table 2.

**Table 2.** Predatory Publications Identified among Plastic Surgery Residency Applications to McGill University between 2015 and 2018 (*n* = 14).

Year published	Journal	Publisher	Impact factor	Days from submission to acceptance	Days from acceptance to publication
2015	JOURNAL OF NEUROINFECTIOUS DISEASES	OMICS International	0	38	42
2015	PLASTIC AND AESTHETIC RESEARCH	OAE Publishing, Inc.	0	108	185
2017	INTERNATIONAL JOURNAL OF PHYSIOLOGY, PATHOPHYSIOLOGY AND PHARMACOLOGY	e-Century Publishing Corporation	0	52	127
2015	ONCOTARGET	Impact Journals	5.008	16	30
2017	AUSTIN JOURNAL OF NUTRITION AND METABOLISM	Austin Publishing Group	0	34	50
2017	JOURNAL OF TUMOR MEDICINE AND PREVENTION	Juniper Publishers	0	8	0
2016	ONCOTARGET	Impact Journals	5.168	58	70
2014	FRONTIERS IN NEUROSCIENCE	Frontiers Media S.A.	3.656	38	64
2016	PLASTIC AND AESTHETIC RESEARCH	OAE Publishing, Inc.	0	195	214
2015	FRONTIERS IN IMMUNOLOGY	Frontiers Media S.A.	5.695	49	63
2014	JOURNAL OF CONTEMPORARY MEDICAL EDUCATION	ScopeMed Publishing	0	N/A	N/A
2012	GLOBAL JOURNAL OF HEALTH SCIENCE	Canadian Center of Science and Education	0	13	80
2012	FRONTIERS IN GENETICS	Frontiers Media S.A.	0	66	72
2015	PLASTIC AND AESTHETIC RESEARCH	OAE Publishing, Inc.	0	72	116
*Mean [IQR]*			0 [0-2.742]	49 [34-66]	70 [50-116]

### Univariate Analysis

Univariate analyses found applicants with a predatory publication differed from other published applicants by a greater number of publications in open access journals (3.29 vs 1.08, *P*-value: .044) (Table 3). There was no significant difference in the median impact factor for publications between applicants who published in a predatory journal and all other published applicants (2.58 vs 3.15, *P*-value = .473).

**Table 3.** Characteristics of Published Applicants by Predatory Journal Publication.

Applicant characteristic	Publication in a predatory journal		*P*-value
	No (N = 103)	Yes (N = 14)	
Applied in 2 or more cycles	5 (4.9%)	0 (0%)	.890
Year of application			
2015	19 (18.4%)	2 (14.3%)	.179
2016	21 (20.4%)	4 (28.6%)	
2017	24 (23.3%)	0 (0%)	
2018	39 (37.9%)	8 (57.1%)	
Male gender	56 (54.4%)	8 (57.1%)	1.000
Medical school region			
Canada	94 (91.3%)	13 (92.9%)	.870
Other	7 (6.8%)	1 (7.1%)	
United States	2 (1.9%)	0 (0%)	
Post-baccalaureate degree	27 (26.2%)	5 (35.7%)	.668
Highest post-baccalaureate Degree			
None	75 (72.8%)	9 (64.3%)	.254
Graduate	24 (23.3%)	3 (21.4%)	
PhD	4 (3.9%)	2 (14.3%)	
Average number of publications (Mean ± SD)	2.87 ± 2.52	6.57 ± 7.21	.079
Average number of publications in an open access journal, (Mean ± SD)	1.08 ± 1.39	3.29 ± 3.69	.044
Average impact factor of publications (Mean ± SD)	3.15 ± 5.72	2.58 ± 2.03	.473

Categorical variables were compared utilizing chi-squared test while means were compared utilizing the Mann–Whitney–Wilcoxon test.

### Logistic Regression

Two separate logistic regression models were constructed to explore the association of applicant characteristics with either having at least one publication in an open-access journal or at least one publication in a predatory journal (Table 4).

**Table 4.** Multiple Logistic Regression Model Describing Applicant Factors Associated With At Least One Publication in an Open Access Journal or a Predatory Journal Compared to Other Published Applicants.

	Applicants with ≥ 1 open access publication		Applicants with a predatory publication	
	OR (95% CI)	*P*-value	OR (95% CI)	*P*-value
Post-baccalaureate degree				
No	Ref	-	Not included	
Yes	0.36 (0.13-0.95)	0.038		
Number of publications				
	1.56 (1.18-1.93)	<.001	1.25 (1.06-1.48)	.010

In the first model exploring characteristics associated with applicants having at least one publication in an open access journal among all published applicants, total number of publications and applicant's having a post-baccalaureate degree were significantly associated with the outcome. Each additional paper published by an applicant increased the odds of having an article published in an open access journal by 1.56 times (95% CI: 1.18-1.93, *P*-value <.001). Applicants who reported a post-baccalaureate degree as their highest level of education had reduced odds of having one article published in an open access journal compared to those with only a bachelor's degree (OR: 0.36, 95% CI: 0.13-0.95, *P*-value = .038).

In the second model exploring applicant characteristics associated with at least one publication in a predatory journal compared to other published applicants, the final model included only one predictive variable: number of publications. For each additional publication, an applicant's odds of publication in a predatory journal increased by 1.25 times (95% CI: 1.06-1.48, *P*-value = .010).

## Discussion

The findings of the current study reveal that 40.2% and 7.5% of the total applicants have contributed to publications in open access and predatory journals, respectively. Applicant characteristics associated with publication in an open access journal included a greater number of publications and one or more post-graduate degrees. Notably, the number of publications reported by an applicant was the only variable positively associated with publication in a predatory journal.

The impact of predatory publishers is important to note from both academic and clinical perspectives. Online dissemination of articles that do not undergo rigorous (or any) peer-review may lead to low-quality research being accessed alongside real science that has been formally evaluated.^17^ This leads to the dilution of the quality and trustworthiness of scientific literature, which may negatively impact both physicians and their patients.^18^ Efforts to attenuate the growth and presence of predatory journals are limited by the absence of well-defined classification criteria. Specifically, there are challenges in evaluating a journal's intent to deceive and identifying the cause of low-quality publications (i.e., insufficient resources vs predatory nature). These challenges are within the context of an overall lack of transparency in the academic peer-review process.^19^ Of note, some populations may be at an elevated risk of being prey to predatory journals, so identifying these populations may enable the development of targeted interventions.

Unfortunately, there is currently an absence of literature on predatory publishing among medical students applying to residency programs. A review of general surgery residency applications in the United States identified 7/643 (1%) journal articles or abstracts listed as peer-reviewed were predatory in nature between 2019 and 2020.^8^ Our paper found a more than 3-fold higher prevalence of predatory publications than this report. The discrepancy between Canadian and American surgical applicants publication in predatory journals may be explained by the heightened competitiveness of plastic surgery residencies in Canada, with the applicant-to-residency ratio ranging from 2.2 to 4.2 between 2015 and 2018 in Canada, which markedly surpassed the 1.4-1.5 ratio observed in the United States during the same period.^20–24^ Moreover, it is plausible that some applicants may have contributed to predatory publications due to a lack of awareness, explaining the correlation of predatory publishing with higher publication count observed in our study. This deficit of knowledge among medical trainees is exemplified by only 7.8% and 23% of medical trainees in Australia or New Zealand, respectively, reporting acquaintance with the term “predatory journal.”^25,26^ Further investigation is needed to elucidate the causes behind medical students’ publication in predatory journals and understand the driving forces behind this phenomenon.

Although there is a scarcity of research reporting the prevalence of predatory publishing among medical trainees, studies exist describing this phenomenon in other populations. In a 2012 sample of 46 000 academics in Italy, almost 5% had published in a predatory journal identified using Beall's list.^27^ Similarly, a review of orthopaedic publications in France identified that 323/6056 (5.3%) were published in potentially predatory journals identified using Beall's list or predatoryjournals.com, while only 0.55% were published in confirmed predatory journals.^28^ These reported prevalences are greater than those seen in our study (3.6%), which may indicate the presence of differing pressures driving these practices among medical trainees in contrast to researchers who are more advanced in their careers.

An interesting finding in our study was that the only applicant characteristics associated with publication in a predatory journal was number of publications. These findings align with recent work that suggests publication pressures may be a key emerging factor increasing use of illegitimate publisher's periodicals.^29^ Our paper adds to existing literature that highlights low experience and working in developing countries as key risk factors for publishing in predatory journals.^6,7^

Notably, three journals included in our study explicitly advertised quick acceptance times, potentially influencing applicants to select these journals during their final year of study to bolster their residency applications. These findings align with prior studies that describe the use of predatory publishing to fast-track promotions among professors within academic institutions as one of many drivers for predatory publishing.^30–32^ While research has identified that medical students believe a history of academic publications will support their match rate to residency, evidence on the value of publications to future match success are mixed. A recent analysis of factors affecting Canadian medical students’ success in the residency match identified no statistical association between number of publications and matching to a first-choice discipline in the first iteration of the match regardless of competitiveness.^33^ Similar findings have been seen in the United States.^34^ Regardless of the impact of publication on applicant competitiveness, honesty, integrity, and ethics are all extremely important characteristics that are required of future residents and affect candidates’ chances of acceptance. Therefore, medical mentors and training programs should stress the importance of these invaluable characteristics to their students, especially young trainees that may experience external pressures to publish quickly from residency programs that include research as a component of their selection criteria.

### Limitations

There are several limitations to consider in the interpretation of this study. It is important to note that, due to the small number of predatory publications in this study, very strong associations would have been necessary to identify the correlation of any applicant characteristics with the outcome. Simultaneously, we did not have access to applicants' ages and therefore could not explore the association of age and our outcomes of interest. For this reason, further studies designed to identify characteristics of student authors publishing in predatory journals will be essential to better understand who is publishing in these settings. Another important limitation in our study design is the absence of a universally acceptable criteria to evaluate the potential predatory nature of a journal.^19^ It is important to recognize when interpreting our results that not all journals listed in Beall's list are necessarily predatory. For example, around 18% of journals from Beall's list that participated in the experiment by Bohannon^35^ did not accept the fake paper sent by the researcher.^35^ As well, 3 articles included in our list of predatory publications, specifically published in Frontiers in Genetics, Frontiers in Immunology, and Frontiers in Neuroscience, are currently listed in the Directory of Open Access Journals (DOAJ) indicating an important discrepancy between our reference indexes. Utilizing a more conservative definition that includes any publications listed on Beall's list and excludes any publications present in the DOAJ, the prevalence of predatory publishing in our study would be 10/388 (2.5%) of all publications. Additionally, as this study was restricted to applicants being considered for an interview at a single institution, our results are not necessarily generalizable to other institutions. Finally, our evaluation of open access publications excluded publications made in “hybrid access” journals defined as journals that provide the option for authors to voluntarily publish a manuscript for an additional fee. This likely led to an underestimation of open access publications in our study.

## Conclusion

Overall, this study highlights a high rate of predatory publication practices among plastic surgery residency applicants in Canada between 2015 and 2018. These findings are important, as they reinforce the need to increase awareness among the plastic surgery community, including medical students, on the deceptive nature of predatory journals. Further research that explores publishing practices among medical trainees will be necessary to clarify potential drivers of these practices.

## Study-Related Presentations

1. Presented at the 2024 Western Medical Research Conference. January 18-20, 2024; Carmel, California.
